# Ageism and (Successful) Digital Engagement: A Proposed Theoretical Model

**DOI:** 10.1093/geront/gnae078

**Published:** 2024-06-14

**Authors:** Ittay Mannheim, Hanna Köttl

**Affiliations:** Department of Communication Studies, Ben-Gurion University of the Negev, Beer-Sheva, Israel; Department of Health Sciences, IMC University of Applied Sciences Krems, Krems an der Donau, Austria

**Keywords:** Ageism, Digital technology, Successful aging

## Abstract

Recently, scholars have highlighted the detrimental consequences of technology-based ageism. Digital technology (DT) is commonly discoursed as an opportunity to promote Successful Aging. Nevertheless, the theoretical implications of ageism, DT, and Successful Aging are underexplored. This paper presents a new theoretical model of Digital Engagement and Ageism (D-EngAge), which elaborates on the potential impacts of ageism on digital engagement and participation in later life and explains how ageism may pose a threat to realizing the potential of DT to promote Successful Aging. The D-EngAge model was developed based on a synthesis of findings from 12 recent studies we conducted on the intersection of ageism and DT. Findings were synthesized through Iversen’s clasification of four dimensions of ageism, demonstrating how ageism as a multifaceted construct has a reciprocal relationship with digital engagement on the micro- (individual), meso- (social interaction), and macro-levels (discourses and societal practices). Consequently, digital engagement on these levels may exacerbate or reduce technology-based ageism. This forum paper identifies ageism as a barrier to utilizing DT, critically discusses power imbalances, and deconstructs Successful Aging discourses regarding digital engagement. Theoretical implications and recommendations for future interventions and policy measures to mitigate ageism and promote digital engagement and participation in later life are presented.

Digitalization and population aging are two major social transformations concerning contemporary modern societies. Over the past two decades, the exponential advancements in digitalization have deluged all areas of everyday life, including work, household, healthcare, and social participation. In an increasingly aging society, digital technologies (DTs) are perceived as a prerequisite for Successful Aging (Wan et al., 2022) and offer various opportunities to age well and grow old in dignity. Rowe and Kahn (1997) make a distinction between “usual” aging and “successful” aging and describe Successful Aging as a continuum across three domains: low probability of disease and disability, high cognitive and physical functional capacity, and active engagement with life. DTs in the context of (successful) aging are, for instance, expected to improve older adults’ access to health services and information (Rush et al., 2022), decrease the burden of healthcare professionals and informal caregivers (Lucero et al., 2019), support cognitive and physical fitness in later life (Irazoki et al., 2020), promote independence and safety, decrease loneliness and social isolation (Shishehgar et al., 2018), and more generally, ensure older persons’ continued active participation in society and everyday life (UNECE, 2021).

Despite the promising nature of DT, recent data highlight that not everyone benefits equally from the potential of DT. In particular, the age-based digital divide remains a challenge on the individual and societal levels (Fang et al., 2019; Friemel, 2016). For example, an analysis by the PEW Research Center stresses that although older persons in the United States increasingly use new DTs in recent years, 25% of those aged 65 and over do not use the internet (Faverio, 2022). Such reports also highlight that chronological age alone does not account for this gap. Disadvantaging factors in accessing DT, such as low educational level or low income, are known to accumulate over the life course, potentially amplifying the digital divide (Fang et al., 2018; Wanka & Gallistl, 2018). Therefore, exploring social, structural, and societal barriers is necessary to foster digital inclusion for individuals of all ages (Gallistl et al., 2020).

Recent evidence has raised awareness about a so far underexplored barrier to DT, namely the phenomenon of ageism. In 2005, a seminal paper by Cutler identified ageism as a potential barrier to realizing digital equity and reducing the digital divide (Cutler, 2005). Previous studies have examined the implications of negative discourse of aging and technology in design (Peine & Neven, 2021; Righi et al., 2017; Vines et al., 2015); addressed potential myths and stereotypes about how older persons use technology (Mitzner et al., 2010; Quan-Haase et al., 2018); and advocated for advancements in the field of aging and technology to promote independence and active aging (Schulz et al., 2015). Nevertheless, only in the past years, scholars have started to empirically explore the potentially bidirectional associations between (self)ageism and DT engagement in later life (e.g., Köttl, Allen, et al., 2022), manifestations of ageism in the design process of DT (Mannheim, Wouters et al., 2023), and other consequences of “digital ageism” (Rosales et al., 2023). Iversen et al. defined ageism as negative or positive stereotypes (how we think), prejudice (how we feel), and discrimination (how we act) against older persons based on their chronological age. Ageism can be implicit or explicit and may come forward on a micro-, meso-, or macro-level (Iversen et al., 2009, p. 15). Ageism is a widespread phenomenon pervading social interactions, institutions, laws, and policies worldwide. It significantly harms individuals’ health, well-being, and dignity, costing society billions of dollars (World Health Organization [WHO], 2015).

Ageism prevents numerous individuals from enjoying their human rights and to reach their full potential. Nevertheless, the negative consequences of ageism in the context of DT are highlighted as an under-researched topic (WHO, 2021), and the strongly discoursed relations between using DT and Successful Aging remain underexplored (Rowe & Cosco, 2016) and unchallenged (Comunello et al., 2023). Visual representations of Successful Aging with DTs portray successful agers as relatively young and active older adults who are highly educated, socially well connected, and capable of mastering new digital devices. In contrast, DTs to mitigate the challenges of aging are often viewed as used by others (e.g., caregivers or healthcare professionals), whereas the older person is not actively involved in the use of the technology (Ivan & Loos, 2023). DT for older persons is, therefore, implicitly associated with perceptions of the fourth age (oldest-old), expecting frailty, cognitive decline, and further age-related losses (Higgs & Gilleard, 2020) while generally framing older adults as late adopters. Negative perceptions of older persons give rise to interventionist approaches to aging and technology, which depict technology as a solution to negative aspects of aging. Such approaches are often adopted by innovation and policy actors and may further amplify stereotypical views of older persons as nonsuccessful agers and detached from technological change (Peine & Neven, 2019). Sociotechnical interventions are a primary focus in research and innovation, particularly in the context of care and healthcare, which can, on the one hand, exacerbate ageist perceptions of DT use and, on the other hand, confront them and empower older persons (Wagner & Ogawa, 2023).

In a rapidly digitalizing world, ageism may, therefore, pose a threat to successful engagement with DT (Köttl, Allen, et al., 2022; Mannheim, Varlamova, et al., 2023; McDonough, 2016; Rosales & Fernández-Ardèvol, 2020), thus limiting the potential of DT to promote so-called Successful Aging. According to the Risk of Ageism Model (Swift et al., 2017), there are three pathways of how ageism may potentially impede Successful Aging, namely through (1) stereotype embodiment, (2) stereotype threat, and (3) age discrimination. Stereotype embodiment refers to internalizing negative stereotypes throughout the life course, shaping views and perceptions towards the aging self. This theoretical model hence argues that as human beings, we internalize negative age stereotypes from the environment that we live in, while these may become self-stereotypes over time (Levy, 2009). Lifelong exposure to an environment that perceives older persons as technophobic or incompetent could lead to lower use of DT and more significant performance problems in older individuals (Köttl et al., 2021). In line with stereotype embodiment, the phenomenon of stereotype threat may also have a self-fulfilling potential concerning age stereotypes, affecting behavior and performance. In situations where individuals feel at risk of confirming certain stereotypes, they may underperform (Steele, 1988). For instance, social situations with seemingly tech-savvy younger adults may provoke stereotype threat experiences and lead to avoidance of using DT (Caspi et al., 2019; Mariano et al., 2020). Stigmatizing elements in technology design may also activate stereotype threat (Köttl et al., 2021; Mannheim, Weiss, et al., 2023). Finally, age discrimination, understood as unequal treatment based on age, could hinder active and successful engagement with DT (Choi et al., 2020). Lack of internet access, DT training opportunities, age limits, patronizing communication, or other discriminatory practices may prevent older adults from engaging with DT (Köttl et al., 2021). This indicates that neither aging nor digitalization is a mere individual process but is a socially constructed phenomenon shaped by culture, societal expectations, and lived experiences (Chonody & Teater, 2017).

The field of aging and technology is often criticized for lacking theory (Wanka & Gallistl, 2018). Accordingly, this paper aims to present a new theoretical perspective on the intersection between aging and DT. We suggest an innovative theoretical model elaborating the bidirectional interaction between ageism and DT engagement in later life based on a synthesis of our recent empirical research. Finally, we discuss the proposed model in light of recent developments in the field of digital ageism, critically reflect on the discourse of Successful Aging with DT, and highlight recommendations for future research and policy measures.

## Digital Engagement and Ageism (D-EngAge): A Proposed Theoretical Model

**Figure 1.**
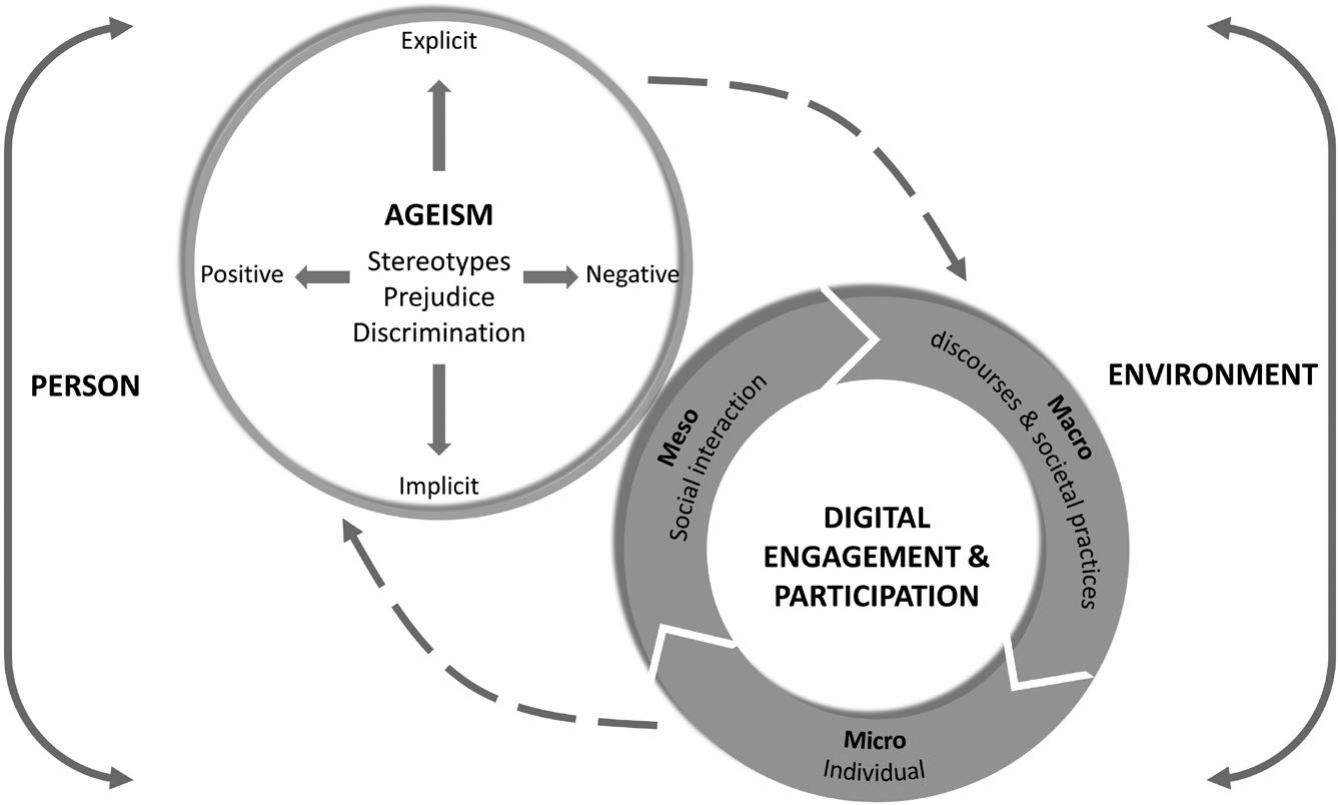
Digital Engagement & Ageism (D-EngAge): a proposed theoretical model.

In the following, we present how the model was structured based on a synthesis of 12 studies conducted over the last 5 years investigating the intersection of ageism and DT. An elaboration of the context, goal, study design, sample characteristics, and analysis of each study is presented in Table 1. First, the general structure of the model is explained (see Figure 1). After that, the model is demonstrated based on the findings, recommendations, and implications of the 12 studies (detailed in Table 2). The synthesis was conducted by triangulating various study designs (literature reviews, qualitative, and quantitative findings), perspectives of different stakeholders involved (older persons, researchers, designers of DT, and healthcare professionals), and linked to the four dimensions of ageism as suggested by Iversen et al. (2009): (1) social–psychological (stereotypes, prejudice, and discrimination) (2) negative–positive valance (3) explicit–implicit representation (4) and micro–meso–macro level. The first three dimensions of Iversen’s definition illustrate the first element of the D-EngAge model, namely, the multifaceted construction of ageism concerning DT. The second element of the model presents the manifestations of ageism regarding digital engagement according to the fourth dimension of Iversens’s definition as occurring in different levels of the individual (micro), social–organizational interaction (meso), and macro-level, targeting discourses and societal practices (e.g., in research, design, media, and policy-making). Notably, the second digital engagement element comprises a dynamic and nonhierarchical movement between micro–meso–macro levels. More so, the two separate elements of ageism and digital engagement interact reciprocally and influence each other in a dynamic and iterative process, interchanging between individuals, design processes, and digital devices and services, as suggested by Peine and Neven (2021). In line with environmental gerontology, emphasizing the person–environment interaction (Lawton & Nahemow, 1973; Moore, 2005; Scharlach & Diaz Moore, 2016), the person and the environment are viewed as interacting and influencing each other, shaping how DTs are used and accepted. This person–environment interaction of digital ageism is reflected in the model through the social, cultural, political, virtual, and physical environments, including social interactions, policies, and media discourses. For example, individual experiences of older persons interacting with a healthcare professional about using a technology-based treatment (social interaction–meso-level) can be influenced by self-ageism of the older person as well as ageist views of the professional (individual-micro-level). The interaction may be positive or negative, subsequently confirming or contrasting age-based stereotypes about how older persons use technology. Consequently, this may influence future perceptions about older persons’ engagement with technology and future interactions between the person and the healthcare professional in the environmental context of receiving technology-based care. In the following, we similarly describe each study and demonstrate how the different facets of ageism reciprocally reflect in the D-EngAge model according to micro–meso–macro levels.

**Table 1.** Detailed Study Characteristics of Synthesized Studies (*n* = 12)

Study	Goal	Study design	Materials and population characteristics	Analysis
Mannheim et al. (2019)	To advocate for the inclusion of older adults in the research and design of DT and provide literature-based guidelines to do so.	Critical ethical literature review	Purposeful and targeted review of literature from 10 years prior to the study, including referrals and consultations with experts in the field of gerontology, gerontechnology, and STS, on what is known about how DT is developed and studied in relation to older adults and studies that discuss social exclusion, ageism, and age stereotypes regarding DT.	Qualitative critical synthesis
Köttl et al. (2020)	To shed light on the temporal reciprocal associations of self-perceptions of aging and everyday ICT usage in later life.	Longitudinal cohort study	Two waves from the German Ageing Survey including community-dwelling German adults aged 40 and older (*N* = 3 600); Mean age 61.78 (*SE* 0.041); 48.9% females; 2.8% with low, 45,1% with medium and 52.2% with high educational level; 29.2% from East Germany. Measurements: Age-Cog Scales addressing the domains physical loss, social loss, and personal competence; Internet use was self-assessed (How often do you use the internet for the following purposes? Contact with friends and relatives, search for new social contacts, search for information, banking business, entertainment, shopping, and creating own contents; 6-item Likert scale from “never” to “daily”)	Structural Equation Modeling (SEM)
Xu and Köttl (2020)	To investigate the interaction between older people’s Internet use and the level of loneliness, as well as to introduce the role that self-perceptions of aging may play in this association.	Cross-sectional design	Wave 6 from the German Ageing Survey including community-dwelling German adults aged over 65 years who had participated in the drop-off questionnaire in 2017 and who had access to the internet (*n* = 2119). Mean age 72.71 (*SD* 5,67); 42.1% females; 28.9% from East Germany. Measurements: Six items version of the De Jong Gierveld Loneliness Scale; Age-Cog scales; Internet use for social contact was assessed by one question (How often do you use the web for being in contact with friends and relatives, e.g., through e-mail, Facebook, chat, video telephony; six-item Likert scale from “never” to “daily”).	Descriptive statistics and pairwise correlations Multiple linear regressions
Köttl et al. (2021)	To explore internalized ageism related to everyday ICT use in older nonusers. To demonstrate how age stereotypes in the context of everyday ICT can be formed and reinforced through disempowering and ageist environments.	Qualitative descriptive design (secondary interview analysis)	Interview transcripts of older adults (65+) living in Austria who have never used a computer and reported not using the internet in the last 7 days (*N* = 15); Age range: 69–88; mean age 79 (69, 88); 66.7% female; 73.3% had achieved postsecondary education (vocational training) or upper secondary education (vocational school); 33.3% lived in rural areas; participants had no known health conditions.	Qualitative content analysis
Mannheim et al. (2021) ^a^, Study a	To assess the explicit and implicit attitudes of healthcare professionals toward older adults’ abilities to use DT and the association between these attitudes and ageism. An additional goal was to develop and test the use of new direct and indirect measurements of DT-related ageism.	Cross-sectional survey	Online survey completed by 97 physiotherapists working in the Netherlands and fourth-year physiotherapy students. Mean age 32.4 (*SD* 11.2); 75% female; 43% had more than 6 years of experience; 47% reported that most of their patients were 65 years and older. Measurements: Fraboni Scale of Ageism (FSA); Attitudes Toward Older Adults Using Technology (ATOAUT); Indirect vignettes measuring attitudes to using different healthcare DTs by different age groups.	Factor Analysis Regression
Mannheim et al. (2021) ^a^, Study b	To test how age salience and stereotype activation might moderate the correlation between general ageism and DT-related ageism.	Cross-sectional experimental design	Online survey completed by 93 healthcare professionals and fourth-year healthcare students in the Netherlands. Age salience (young vs old) was manipulated. Mean age 37.01 (*SD* 11.9); 67% female; 49% had more than 6 years of experience; 41% reported that most of their patients were 65 years and older. Measurements: Expectations Regarding Ageing (ERA-12); ATOAUT; Indirect vignettes measuring attitudes to using different healthcare DTs.	Regression Moderation
Köttl, Allen, et al. (2022)	To understand the exact nature of the associations between everyday ICT usage and (self-)ageism as well as potential moderators.	Systematic literature review	A systematic search was performed in eight academic databases (January 1995 to January 2021). The review followed the PRISMA guidelines. Fifteen articles were included in the analysis.	NHLBI quality assessment tools for risk bias Narrative synthesis
Köttl, Tatzer, et al. (2022)	To critically examine media portrayals of older adults’ everyday ICT usage during the first and second wave of the COVID-19 pandemic.	Qualitative discourse analysis of media articles	Using the LexisNexis database, a search of three of the five most circulated German newspapers was completed. Namely, die Sueddeutsche Zeitung, die Frankfurter Allgemeine, and Die Welt. The search was limited to newspaper publications from March 11, 2020, to December 1, 2020.	Discourse analysis and thematic analysis
Mannheim, Wouters et al. (2023)	To address what are the explicit and implicit manifestations of ageism in the design process of DTs intended for the use of older persons. To identify how ageist discourse is embedded in the design process, and how it may influence the involvement of older persons.	Scoping review	Seven databases were screened for studies reporting on the design of DT with older persons between January 2015 and January 2020. The review followed the PRISMA-scr guidelines. Sixty articles were included in the analysis.	Critical Discourse Analysis (CDA)
Mannheim, Varlamova, et al. (2023)	To investigate whether ageism and negative Attitudes Toward Older Adults’ Abilities to Use Technology (ATOAUT) may moderate the intention to use and actual use of DT by older persons within the theoretical framework of the UTAUT-2 model.	Cross-sectional survey	374 persons aged 50 and above from the Netherlands filled out a survey online (77%) or on paper (23%). Age ranged from 50 to 97, mean age 69.3 (*SD* 8.6); 47.6% female; 57% were retired; 56.2% resided in cities, 69.8% were married or living with a partner; 52.3% had a higher education; and 76.1% indicated that their health does not limit their daily activities. Measurements: ATOAUT, ERA-12, Experienced ageism, Technology acceptance (UTAUT-2)	Structural Equation Modeling (SEM)
Mannheim, Weiss, et al. (2023)	To investigate manifestations of ageism in the design process of DT through the perspectives of older persons who participated in co-designing DTs about the design process, aging and technology, their role in the design process and the interaction with the designers.	Focus groups	Twenty-one older adults who were part of a community of persons co-designing DTs within an age-tech innovation hub, participated in three focus groups. Age ranged from 59 to 79, mean 68.8 (*SD* 5.3); 57% female; retired between a few months to 13 years; physically independent, community dwelling from a city in southern Israel.	Thematic analysis
Neiertz et al. (2023)	To investigate whether ageism and negative attitudes towards older adults’ abilities to use DT may influence the decisions of physiotherapists to use healthcare-related DT with older adults. Furthermore, to investigate additional personal and social characteristics that may influence physiotherapists’ attitudes toward using DT with older adults.	Cross-sectional survey	Online survey completed by 78 physiotherapists working in Luxembourg. Age ranged from 23 to 64, mean 38.7 (*SD* 9.8); 51.3% female; Mean work experience 14.1 years; 59% worked with 50% or more patients older than 50 years. Measurements: Attitude towards technology use in the work environment, ATOAUT, ERA-12, use of DT in treatments.	Logistic regression

*Notes*: ATOAUT = Attitudes Toward Older Adults Using Technology; CDA = critical discourse analysis; DT = digital technology; ERA-12 = Expectations Regarding Aging; FSA = Fraboni Scale of Ageism; ICT = Information and Communication Technology; PRISMA = Preferred Reporting Items for Systematic Reviews Meta-Analyses; *SD* = standard deviation; *SE* = standard error; SEM = Structural Equation Modeling; STS = Science and Technology Studies; UTAUT = Unified Theory of Acceptance and Use of Technology.

^a^ Mannheim et al. (2021) consisted of two studies.

**Table 2.** Results, Recommendations, Implications, and Model Components of Synthesized Studies (*n* = 12)

Study	Main results	Recommendations and implications	Ageism components	Level of influence on digital engagement
Mannheim et al. (2019)	Exclusion from research and design processes of DT were identified; Aging was found to be stereotypically framed as a problem that needed to be fixed, and older adults related to as frail and incompetent; Subsequently, many of the technologies developed for the use of older adults focus on care.	Exclusion was identified as a form of ageism in research and design. Nevertheless, mere inclusion does not assure ageism-free research and design. Main ethical considerations and guidelines identified and suggested for inclusive participation included: awareness of stereotypes and ageism; consent and re-consent; autonomy, trust and respect; research methods and tools; safety and security	Stereotypes, prejudice, and discrimination	Macro—research, design, and policy related to DT
Köttl et al. (2020)	Low everyday ICT engagement preceded more negative self-perceptions of aging related to personal competence 3 years later: *B* = –0.06; *SE* = 0.02; *p* < .001. The association between self-perceptions of aging and everyday ICT engagement was, however, nonsignificant.	This study calls for inclusive everyday ICT design and policy-making that enables older adults to access and adopt important everyday ICT. It recommends participatory and co-constitutional design approaches and interventions to promote lifelong learning and contribute to ageism-free environments.	Stereotypes and prejudice; implicit; negative ageism	Micro—self-ageism
Xu and Köttl (2020)	Higher Internet use was negatively correlated with the feeling of loneliness (*r* = −0.07, *p* < .01), while more positive self-perceptions of aging (SPA) were moderately correlated with a lesser feeling of loneliness (*r* = −0.42, *p* < .001). SPA (in the aspect of personal competence) moderated the association between Internet use and loneliness (*B* = −0.02; *SE* = .01; *p* = .04).	This study suggests on the one hand, to empower older adults with negative self-perceptions of aging to ensure greater access to ICT and on the other hand to challenge the prevalent stereotypes about older people and aging in societies through targeted interventions.	Stereotypes and prejudice; implicit; negative ageism	Micro—self-ageism
Köttl et al. (2021)	Two major categories were identified: (1) age stereotypes related to everyday ICT use in later life and (2) environmental cues contributing to the activation and internalization of age stereotypes; within these categories four thematic frameworks were identified: relevance and use, competence and learning, design of technology, and intergenerational contact. Older participants attributed nonuse of ICT to their older chronological age and portrayed themselves as lonelier, less active, technology- refusing, incompetent, incapable or cognitively declining. Ageist social interactions and intergenerational comparisons as well as disempowering and stereotypical features and aspects in the technology design were found to contribute to the narrative of the “older nonuser.”	The findings of this research call for participatory everyday ICT design, ageism-free everyday ICT learning contexts, and greater awareness about lifelong learning on the political and societal level to overcome ageism as a barrier to everyday ICT.	Stereotypes; prejudice, and discrimination; explicit and implicit; negative ageism	Micro—self-ageism; Meso—social interactions with family members; Macro—technology design
Mannheim et al. (2021) ^a^, Study a	Factor analysis of the ATOAUT scale identified 10 items (out of 15) with high loadings. The Cronbach α of the 10-item scale was 0.82 and explained 91.2% of the variance of the 15-item scale. Physiotherapists rated older adults as young as 50 years as less able to use healthcare DT described in the vignettes compared to younger adults (*p* < .001). A multiple regression analysis revealed that higher levels of ageism, beyond other predictors, were predictive of more negative ATOAUT, (β = 0.36; *t* = 3.7; *p* < .001).	Ageism is identified as a potential barrier to adoption of DT in healthcare. The use of indirect measures (vignettes) and direct measures (ATOAUT scale) was found to be useful to identify DT-based ageism. Future studies should broaden the validation of the ATOAUT-10 scale on more diverse samples and focus on the discriminatory aspect of ageism and self-ageism of older adults.	Stereotypes and prejudice; explicit and implicit; negative ageism	Meso—social interactions with healthcare professionals
Mannheim et al. (2021) ^a^, Study b	Younger adults were perceived as more likely to use the described DTs compared to older adults. This was qualified by a significant regression model (adjusted *R*^2^ = .59; *p* < .001), with the age salience condition being the only significant predictor (β = –0.78; *p* < .001). An additional moderation analysis found that old age salience moderated the correlation between ageism and ATOAUT (*R*^2^ = 0.19; *p* < .01), such that higher levels of ageism correlated with more negative ATOAUT in the old age salient condition, but not the young condition.	Activation of age-based stereotypes may occur implicitly and may play a pivotal role in adoption of healthcare DT. The effect sizes found were relatively low, suggesting that future studies should test stronger manipulations of stereotype activation and take into account additional characteristics. It is suggested that in order to increase adoption of DT, the focus ought to shift toward how we can change stereotypes and their activation through raising awareness and training programs for healthcare professionals.	Stereotypes and prejudice; explicit and implicit; negative ageism	Meso—social interactions with healthcare professionals
Köttl, Allen, et al. (2022)	Fifteen studies were included, exploring stereotype embodiment (*n* = 9), stereotype threat (*n* = 3), and age discrimination (*n* = 3); Self-ageism was found to be associated with ICT use, participation in training, and performance. Stereotype threat may predict everyday ICT usage over time while everyday ICT itself may provoke stereotype threat in older persons. Age discrimination (e.g., paternalistic talk) was found to negatively influence use and performance.	This review calls for “train-the-trainer” initiatives to combat ageism in technology learning settings as well as targeted interventions to address (self-)ageism in older adults to enhance their ICT use and empower them. Moreover, participatory and inclusive technology design initiatives should be forwarded to decrease ageism in the design of ICT.	Stereotypes, prejudice, and discrimination; explicit and implicit; negative ageism	Micro—self-ageism, perceived age discrimination; Meso-level—social interactions with ICT educators and with students
Köttl, Tatzer, et al. (2022)	Fifty-one included articles were identified: interviews (15.7%), letter to the editor (3.9%), general articles (41%); Article tone: overly positive (43.1%), overly negative (27.5%), neutral or mixed (27.5%); Three major themes (a) (non-)stereotypical images of aging and everyday ICT use, (b) aging actively in the digital era, and (c) everyday ICT use is power. Media discourses varied from constructing stereotypical and dichotomous images of aging to the depiction of older persons as overly active technology users and (future) consumers. Few articles presented a nuanced portrait of aging in the digital era, emphasizing the heterogeneity of older people.	These findings encourage researchers, technology developers, and policy-makers to promote participatory research and ICT design initiatives to enable barrier-free and ageism-free ICT usage. Moreover, journalists are called to include older persons as sources of primary account in their reporting to avoid power imbalances and stigmatization.	Stereotypes and prejudice; explicit and implicit; negative and positive ageism	Micro—self-ageism; Meso—social interactions with family members; Macro—technology design, media and policy discourses
Mannheim, Wouters et al. (2023)	Various explicit and implicit forms of exclusion of older persons from the design process were identified, such as no or low involvement, upper-age limits, and sample biases toward relatively “active,” healthy and “tech-savvy” older persons. Critical discourse analysis revealed the use of outdated language, stereotypical categorizations, and/or design decisions based on ageism in 71.7% of the studies. A discrepancy was found between an “ideal” discourse regarding the involvement of older persons throughout the design process and actual practice.	This study calls for greater awareness on how ageism influences the design process of DT. Involvement per se does not immediately account for a better design outcome. Meaningful involvement and partnership with older persons can facilitate better design outcomes and reduce negative discourse. The results are limited to what was visible in the articles. Future studies should critically observe aging discourses in actual design processes. Potential solutions were identified in the articles to overcome design errors and biases of exclusion, user involvement, discourse on aging, and speed versus quality.	Stereotypes; prejudice, and discrimination; explicit and implicit; negative and positive ageism	Macro—influence on design of DT
Mannheim, Varlamova, et al. (2023)	More negative ATOAUT was found to correlate with higher ERA, higher experienced ageism, and lower perceptions of all technology acceptance factors except for social influence (*r*’s between 0.18 and 0.41). A path analysis found that adding ageism to the UTAUT-2 model slightly improved fit according to the RMSEA. *R*^2^ of Behavioral Intention increased to 0.783. The association between older age and more negative levels of ATOAUT was mediated by higher levels of ERA-12 (Indirect effect β = 0.08, *p* < .001). Negative ATOAUT (rather than age) moderated the associations of effort expectancy, facilitating conditions, and habit with behavioral intention to use technology.	Self-ageism needs to be addressed in interventions to increase the use of digital technology by older persons. Raising awareness of ageism in the context of DT may assist professionals, policy-makers, and technology designers in empowering older persons and reducing stereotypical assumptions about the ability of older persons to use DT. Future studies should attempt to include as diverse as possible samples to test the role of ageism on technology acceptance, including multinational longitudinal surveys, and investigate specific use cases of DT.	Stereotypes; prejudice, and discrimination; explicit and implicit; negative ageism	Micro—self-ageism; Meso—experienced ageism from others
Mannheim, Weiss, et al. (2023)	While not asked directly, ageism was described as experienced by participants in their daily lives and interactions with the designers during the design process. Manifestations of ageism were strongly identified in an intergenerational context of younger persons (mainly family members and the designers) versus older persons. Negative images of aging were pointed out as a potential influencing factor on design decisions.	This study highlights the potential role of ageism as a detrimental factor in how DTs are designed. Positive experiences of inclusive design pointed out the importance of creating a “partnership” in the design process. Participants defined the “ultimate partnership” in codesigning as processes in which they were involved from the beginning, iteratively, in a participatory approach. Such processes were perceived as leading to successful design outcomes, which they would like to use, and can reduce intergenerational tension.	Stereotypes; prejudice, and discrimination; explicit; negative and positive ageism	Micro—self-ageism; Meso—social interactions with family members and designers of DT; Macro—influence on design of DT
Neiertz et al. (2023)	30.8% of the physiotherapists reported that they had, in the past, not offered DT-based treatment to a patient because of the patient’s older age. Using logistic regression, negative ATOAUT was found to predict not offering technology-based treatment, such that participants with more negative ATOAUT (1 *SD* higher) were 2.17 times more likely not to offer treatment (*p* < .01). Positive attitudes towards using technology in the work environment were also found to be a significant predictor. All other predictors were not significant (Nagelkerke *R*^2^ = .226).	This study demonstrates that technology-specific ageism may lead to discrimination and deprive older persons of optimal treatment. It is important to ensure that the education of physiotherapists and allied healthcare professionals raises awareness to this phenomenon. In addition, it is also important to offer experienced physiotherapists the opportunity to train in the use of emerging technologies, so they are knowledgeable on how to use them with patients of any age. More research is needed to identify the magnitude of ageism in using technology-based treatment with additional healthcare professionals and develop interventions to overcome it.	Stereotypes; prejudice, and discrimination; explicit; negative ageism	Meso—social interactions with healthcare professionals

*Notes*: ATOAUT = Attitudes Toward Older Adults Using Technology; CDA = critical discourse analysis; DT = digital technology; ERA-12 = Expectations Regarding Aging; ICT = Information and Communication Technology; PRISMA = Preferred Reporting Items for Systematic Reviews Meta-Analyses; RMSEA = root-mean-squared error of approximation; *SD* = standard deviation; *SE* = standard error; SEM = Structural Equation Modeling; SPA = self-perceptions of aging; UTAUT = Unified Theory of Acceptance and Use of Technology.

^a^ Mannheim et al. (2021) consisted of two studies.

### Micro-level

Over the past years, we have examined ageism on the micro-level, in particular, the bidirectional associations between (self-)ageism and DT usage in various studies. In a systematic literature review by Köttl, Allen, et al. (2022), we synthesized scholarly evidence on the associations between everyday DT usage, (self-)ageism, and potential moderators. Sixteen studies were identified, indicating significant associations between DT use and stereotype embodiment, stereotype threat, and age discrimination. Thus, providing evidence of manifestations of ageism on the individual-micro-level, but also experienced ageism on the social interaction–meso-level. Notably, additional individual characteristics such as the role of age (group), gender, and motivation were found as potential moderators. Although the systematic literature review supported both directionalities, most studies examined the influence of self- and other-directed ageism on DT use in later life. The review highlighted the importance of positive subjective aging perceptions to engagement with DT in later life while emphasizing the detrimental consequences of ageism in DT learning settings and technology design on older persons’ willingness and ability to use new technology in older age.

Comparably, a qualitative study based on semistructured interviews, aiming to reveal internalized ageism in technology-alienated older adults, demonstrated devaluations of the older self, low self-efficacy, experienced performance challenges, and disengagement from important DTs due to older age, constituting an invisible barrier to successful DT engagement in later life (Köttl et al., 2021). Study participants attributed the nonuse of DT to their older chronological age and described themselves as more socially isolated, less active, technology-refusing, incompetent, or cognitively declining. Even though participants mentioned physical environmental barriers, including the complexity or the inconvenient haptic of the digital device, they overly placed responsibility for not engaging with DTs onto themselves, their chronological age, and age-related losses, which points to the power of the physical environment in activating DT-related age stereotypes and inducing stereotype threat.

Similar findings were drawn from a focus group study involving relatively tech-savvy older adults (Mannheim, Weiss, et al., 2023), emphasizing the role of DT use in promoting positive emotions of joy and pride, and motivation for lifelong learning. On the other hand, negative emotions, such as fear and shame, were found to constitute a barrier to the use of DT. Fear of breaking the DT or making irreversible mistakes and comparisons with younger “fearless” adults were prominent. Also, the shame of appearing weak and incompetent in intergenerational interactions due to low digital skills was prevalent. Thus, the mentioned shame and fear were identified as a form of stereotype threat leading to avoidance of using DT.

Drawing on survey data and employing a cross-lagged model, Köttl et al. (2020) have also explored the temporal reciprocal associations of self-perceptions of aging and DT use in later life, aiming to understand whether internalized ageism affects DT engagement or whether it is nonuse that increases self-ageism over time. Their findings indicated that low DT engagement in older adults preceded more negative self-perceptions of aging related to personal competence 3 years later. In an additional study, Xu and Köttl (2020) found a moderating role of positive self-perceptions of aging on the association between higher internet use and lower levels of loneliness among older persons.

Furthermore, Mannheim, Varlamova, et al. (2023) have investigated self-ageism and technology-related ageism within the framework of the Unified Theory of Acceptance and Use of Technology (UTAUT-2; Venkatesh et al., 2012). A path analysis found that negative expectations regarding aging partially mediated the association of chronological age with negative attitudes toward technology use. This suggests that people aging with more negative old age stereotypes might be more prone to adopt negative perspectives on how older persons (should) use technology. Significantly, negative technology-based ageism moderated the associations of several influencing factors of the UTAUT model (effort expectancy of using DT and facilitating conditions such as help from others) on the intention to use DT. Particularly, facilitating conditions, which include the ability to receive help from others and the interaction with technology-based ageism, were strongly associated with the intention to use DT. These findings challenge stereotypical conceptions that higher chronological age is associated with lower intention to use DT and emphasize the role of individual-level ageism, on the one hand, and positive social interactions on the other to facilitate digital engagement.

### Meso-level

Social interactions play a crucial role when exploring the role of ageism in the context of DT usage in older age (Luijkx et al., 2015; Xi et al., 2022). Mannheim, Varlamova, et al. (2023) found that receiving help from others was a prominent factor in digital engagement. The perspective of older persons involved in the design process of DT revealed that negative images of aging and ageism were experienced in their daily lives with family members (viewing older persons as lagging behind) and interactions with the designers during the design process (imagining them as frail and less capable of using new DT; Mannheim, Weiss, et al., 2023). Such ageist experiences were described as verbal and direct, as well as nonverbal, such as talking aggressively or impatiently. Intergenerational interaction was occasionally described as positive (ability to learn from younger persons) or negative (dependency on others to learn). Congruently, in Köttl et al. (2021), learning to use a new DT was strongly associated with intergenerational support. Nevertheless, disempowering or ageist practices were highly prevalent in intergenerational learning contexts. Stereotypical assumptions and expectations were widespread in participants’ younger family members, for example, that older persons are less capable of engaging with DTs or that teaching an older adult to use a new DT requires much patience. Furthermore, lack of support, verbal aggression, devaluation, the feeling of being left alone with the DT, and being placed in a waiting and dependent position to receive support contributed to nonuse and highlighted the potential for unequal power relations across generations.

In three additional studies, explicit and implicit measures of stereotypical and prejudiced Attitudes Towards Older Adults’ Abilities to Use Technology (ATOAUT) were identified as a possible barrier and moderator of how healthcare professionals view older adults’ actual use of DT in a healthcare environment. In Mannheim et al. (2021, Study a), physiotherapists rated older adults as young as 50 as less able to use healthcare-related DT. Higher levels of general ageism were associated with more negative ATOAUT. In Mannheim et al. (2021, Study b), healthcare professionals’ perception of chronological age was manipulated. Old age salience, implicitly manipulated, was found to moderate the correlation between ageism and ATOAUT. Hence, higher levels of ageism correlated with more negative ATOAUT in the old age salient condition but not the young condition. These studies suggest that negative perceptions of old age and DT may be implicitly influenced by environmental cues and influence how professionals use or do not use DT to assist or improve older persons’ quality of life. Notably, Neiertz et al. (2023) found that negative ATOAUT predicted physiotherapists’ actual decisions of not offering digital healthcare to a person in the past because of their age. The latter finding is unique as it is currently one of the only documented studies demonstrating that technology-based stereotypes and prejudice may eventually lead to actual technology-based discrimination.

### Macro-level

Ageism on the micro- and meso-levels may eventually affect the macro-level discourses and societal practices in designing digital services and products, media representations, and policy-making (and vice versa). In Mannheim et al. (2019), a critical ethical literature review, the exclusion of older persons from the research and design of DT was identified as an ethical concern. Importantly, this form of discrimination seemed to be fueled by considering older persons as frail, incompetent, and vulnerable. Whereas DTs, focusing mainly on care and healthcare applications, are designed as solutions for the “problems” of aging (Neven & Peine, 2017), without the perspectives of the older persons who are supposed to benefit from them. Thus, a gap is revealed between what (and how) technologies are researched and developed and what older persons want and need. In the scoping review by Mannheim, Wouters et al. (2023), a critical discourse analysis (CDA) of 60 articles identified explicit and implicit manifestations of ageism in the discourse and practice of designing DTs for older persons. Surprisingly, a discrepancy was found between acknowledging the “ideal” practice of involving older persons throughout the design process, as emphasized by almost all studies, and their actual practice of limited involvement. Beyond the complete exclusion of older persons in some studies, several additional forms of exclusion from the design process were identified, such as shallow involvement compared to other stakeholders; involvement commonly taking place only in the first instances of the design process or testing phase, but not in the actual prototype development; and upper-age limits and sample biases toward relatively active, healthy, and tech-savvy older persons. Alarmingly, the CDA also highlighted that 71% of the analyzed studies used outdated language and stereotypical categorizations, which often altered design decisions. This indicates that ageism may compromise research and the design of DT that is targeted explicitly at older persons.

The findings from these reviews converged with those in Mannheim, Weiss, et al. (2023), with a community of older persons who participated in previous design processes of DT within an innovation hub. Participants in this study portrayed an intergenerational gap in how they are initially and stereotypically imagined as lagging behind as potential users and participants in the design process by the (young) designers and entrepreneurs. Similar to findings from Mannheim, Wouters et al. (2023), participants linked the designers’ negative perceptions of aging to how older persons were involved in the design process and eventually to the designed product’s appeal. Importantly, positive experiences of inclusive design in which the participants were iteratively involved from the beginning and throughout the design process in a participatory approach led to a definition of an “ultimate partnership.” From their perspectives, such a partnership reduced intergenerational tension, changed stereotypes, and resulted in designing DTs they would like to use. This finding was also found in Mannheim, Wouters et al. (2023) in articles with positive discourse (no ageism) and higher levels of involvement, which also reported higher satisfaction with the design outcome. Thus, indicating a potential reciprocal and interventional benefit from intergenerational co-design processes.

In an additional study applying CDA of news media on older adults’ DT engagement, Köttl, Tatzer, et al. (2022) have also warned about the potential role that media and policy discourses play in perpetuating ageism and carrying further the image of the “older non-user” or the older “digital immigrant.” Although the findings of this CDA and thematic analysis highlighted a nuanced portrait of aging in the digital era, emphasizing the heterogeneity of older adults, stereotypical and dichotomous images of aging and DT use were also widespread. In addition, media discourses often leaned toward a neoliberal and Successful Aging rationale, placing nonuse onto older individuals instead of acknowledging the variety of socioeconomic factors for low DT use.

## Discussion and Future Agenda

This paper aimed to introduce a new model explaining the complex reciprocal interaction between ageism and DT engagement of older persons and to critically discuss the interventionist assumption that technology can promote Successful Aging. As demonstrated in the D-EngAge model, the associations between the multifaceted construct of ageism and DT engagement and participation are bidirectional. Subsequently, the D-EngAGE model points out the importance of viewing ageism as a multifaceted phenomenon strongly intertwined with the environment, including virtual, physical, social, cultural, economic, and political environmental factors. Importantly, interactions on the social–organizational (meso)-level and macro-level, including discourses and societal practices, can amplify or reduce ageism at the micro-individual level. Negative or positive internalized ageist perceptions may then further exacerbate negative aging discourses and ageist environments.

### Tackling Successful Aging Discourses Regarding Digital Engagement

The cumulation of evidence indicates that besides negative aging discourses related to technology use, positive aging perceptions significantly shape and perpetuate technology-related ageism (Köttl et al., 2021; Köttl, Tatzer, et al., 2022). The terminology of Successful Aging, Active Aging, Productive Aging, or Healthy Aging (WHO, 2002; Rowe & Kahn, 1997) is widespread in popular media and extensively discoursed in (inter)national policies and research (Asquith, 2009). With regards to DT engagement, this is often framed as the potential of DT to increase older persons’ independence, safety, and activity levels and, quite generally, to facilitate aging in place and improve their quality of life (Schulz et al., 2015). The original thoughts of Rowe and Kahn (1997) were that the framework of Successful Aging could assist in reducing ageism and divert the focus from frailty by focusing on psychosocial factors and personal responsibility and agency. Nevertheless, their approach was often criticized for encouraging discourses that have the power to create normative expectations about later life and influence the notion of “aging well” on the individual and societal level (De São José et al., 2017; Rubinstein & de Medeiros, 2015). Positive aging or “aging well” is, however, commonly framed as an individual decision or the individual’s responsibility, neglecting the different starting points people have based on social, educational, or socioeconomic backgrounds (Rudman & Molke, 2009) and diversity in lifestyle and daily routine decisions (Calasanti & King, 2021). The D-EngAge model suggests deconstructing the positive (albeit often paternalistic or interventionist) framing of aging discourses in the context of DT engagement in later life. Accordingly, as identified in several studies presented in this paper, technologies for older persons tend to focus on assistive technologies or care-related DT to support Successful Aging. In contrast, successful engagement with technology is critically viewed within an intergenerational lens as coping with a digitalizing environment. In the context of DT, active engagement is often framed as based on a person’s life choice and motivation, neglecting the unequal access older persons experience regarding DTs (EU Fundamental Rights Agency, 2023) or the implicit biases that may perpetuate digital engagement.

Notably, the views of older persons on what it means to age successfully differ from those of researchers and policymakers and focus on broader aspects such as social inclusion, social relationships and interactions, and engagement with “life” (Teater & Chonody, 2020). While the Successful Aging agenda aims at maintaining high functional levels, avoiding disease, and generally staying active (Rowe & Kahn, 1997), the aging and innovation discourse, widely adopted in policy and practice, emphasizes a rhetoric associating older persons mainly with negative aspects of aging, frailty, cognitive decline, and dependency (Neven & Peine, 2017). Accordingly, findings from the literature (e.g., Mitzner et al., 2010; Quan-Haase et al., 2018) and our studies point to a diversity of motivations and needs of older persons about the potential of DT to enhance their social participation and well-being. Thus, from the D-EngAge point of view, the current aging and innovation discourse primarily focuses on stereotypical assumptions of “non-successful aging.”

On the policy level, positive aging discourses fit well with neoliberal political approaches, aiming to reduce costs and promote privatization (Rudman & Molke, 2009). Although this rhetoric successfully directs significant investments to solve so-called problems of aging, it also steers innovation toward technologies that are not useful for most older persons (Neven & Peine, 2017), thus affecting their adoption (Greenhalgh et al., 2017). In their critical paper on Successful Aging 2.0, Calasanti and King (2021) recommend a shift from fixating on the medical conditions resulting from aging and referring to old age as a problem and instead emphasize diversity and age relations. Congruently, in the D-EngAGE model, we argue that internalized ageism and ageism of others, rather than chronological age, may result in lower use of DT and catalyze a cycle of designing technologies focusing on solving so-called problems of aging rather than focusing on what older persons find beneficial for meeting their needs and improving their quality of life.

Katz and Marshall (2004) argue that positive aging discourses also tend to fuel a certain binarity of older age, categorizing older persons into “functional” and “dysfunctional.” This becomes evident in the context of DT use. For methodological reasons, researchers often employ dichotomous distinctions of study participants into users/nonusers or active/inactive technology users (Köttl et al., 2021), which may amplify marginalization and “othering” (Pickering, 2001). Furthermore, earlier research has indicated that media discourses on older adults and DT use mainly contribute to overly positive images of aging, portraying healthy, active, and consuming older persons (Gallistl et al., 2020; Ivan & Loos, 2023) while neglecting the diversity of older adults. It may also be argued that there is no such thing as a nonuser because technology is embedded in many everyday activities today and is, therefore, unavoidable (Köttl et al., 2021). Hence, the D-EngAGE model promotes a multidimensional conceptualization of DT engagement, avoiding binary distinctions between users and nonusers.

### Digital Ageism as Reflected in Power Relations

Age may be perceived as a power structure in the digital era that intersects with other injustices and shapes us on the societal and individual levels (Calasanti & King, 2021; Krekula, 2009). This becomes obvious concerning who gets a voice in public discourses regarding DT and aging (e.g., Köttl et al., 2021) or who is included in research about technology design (Mannheim, Wouters et al., 2023). In the context of technology adoption, this comes forward in the strong policy focus on data focusing on the usage of technological devices and the internet (Fernández-Ardèvol & Grenier, 2022) and interventions on the individual level (e.g., digital literacy programs; Asquith, 2009; Gallistl et al., 2020; Rudman & Molke, 2009). Rather than addressing unequal access to DT at a broader meso- or macro-level (e.g., promoting inclusive and ageism-free technology design or combatting ageist discourses). Notably, while assuring access to DT is by itself an important goal, focusing on promoting partnerships to facilitate creative digital skills is essential to promoting agency and active digital participation in society (Reuter et al., 2023), rather than the currently established perception of older persons as passive and vulnerable users of DT.

## Conclusion and Recommendations

This paper introduced the D-EngAGE model and demonstrated via the triangulation of recent studies the plausible manifestations and influences of ageism on digital engagement and participation. Ageism as a multifaceted construct can influence digital engagement on the micro-individual, meso-social interaction, and macro-level discourses and societal practices. These levels are dynamic and interact with one another and may have a reciprocal relationship with the construct of ageism, consequently exacerbating or reducing technology-based ageism. Therefore, creating interventions and policies to mitigate this cycle may have promising implications for reducing ageism and increasing digital engagement. Based on our synthesis of studies, we suggest the following recommendations: (1) Change the negative discourse on aging through awareness-raising and training. Raising awareness is essential for people of all ages, but particularly for younger persons who are also healthcare professionals, designers, and generally the future older persons. (2) Empowerment of older persons through designated interventions. It is vital that such interventions also aim to include people who are considered more at risk of being digitally excluded, namely those with lower education and income, minorities, or refugees. Such interventions should be tested and evaluated in research (including a more diverse cultural lens). (3) Aim for inclusion and partnership in the design of digital products and services and design for versatile needs of older persons (not only care and healthcare). Policy-makers have a significant role in making sure funded projects are inclusive. (4) Further develop implicit and explicit measurements and investigate the manifestations of technology-based ageism in additional contexts (e.g., in social assistive robots, data, AI applications, and the workforce). Finally, (5) continue to explore the contribution of DT to Successful Aging and voice the perspectives of older persons on what constitutes the meaning of Successful Aging with DT for them.

Based on the recent cutting-edge research on ageism and DT in the last 5 years, we believe that the digital divide will not naturally dissolve itself as more technological-savvy cohorts start to age. Failing to address technology-based ageism as a barrier to the successful implementation of DT, as recommended earlier, may even widen the digital divide as technology is exponentially developing and evolving. On the other hand, successfully addressing technology-based ageism in policy, research, and practice may empower future older persons and increase digital engagement that can successfully promote older persons’ social participation, well-being, and quality of life.

## Data Availability

The authors do not report data, and therefore, the preregistration and data availability requirements are not applicable. All studies by the authors elaborated in the current model have been previously published. More information about the data used may be viewed in the publications or upon request from the authors.
